# Long-term utilization and benefit of luspatercept in transfusion-dependent, erythropoiesis-stimulating agent-refractory or -intolerant patients with lower-risk myelodysplastic syndromes with ring sideroblasts

**DOI:** 10.1038/s41375-023-02031-7

**Published:** 2023-09-26

**Authors:** Uwe Platzbecker, Valeria Santini, Rami S. Komrokji, Amer M. Zeidan, Guillermo Garcia-Manero, Rena Buckstein, Dimana Miteva, Karen Keeperman, Natalia Holot, Jose Alberto Nadal, Yinzhi Lai, Sadanand Vodala, Barbara Rosettani, Ana Carolina Giuseppi, Aylin Yucel, Pierre Fenaux

**Affiliations:** 1 Medical Clinic and Policlinic 1, Hematology and Cellular Therapy, University Hospital Leipzig, Leipzig, Germany; 2 MDS Unit, AOU Careggi, University of Florence, Florence, Italy; 3 Moffitt Cancer Center, Tampa, FL USA; 4 Department of Internal Medicine, Yale School of Medicine and Yale Cancer Center, Yale University, New Haven, CT USA; 5 Department of Leukemia, The University of Texas MD Anderson Cancer Center, Houston, TX USA; 6 Odette Cancer Centre, Sunnybrook Health Sciences Centre, Toronto, ON Canada; 7 Celgene International Sàrl, a Bristol-Myers Squibb Company, Boudry, Switzerland; 8 Bristol Myers Squibb, Princeton, NJ USA; 9 Service d’Hématologie Séniors, Hôpital Saint-Louis, Assistance Publique-Hôpitaux de Paris and Université Paris 7, Paris, France

**Keywords:** Randomized controlled trials, Anaemia, Myelodysplastic syndrome

## To the Editor:

Myelodysplastic syndromes (MDS) are disorders of hematopoietic stem cells characterized by ineffective hematopoiesis, commonly leading to anemia [1]. The main goals in the treatment of lower-risk MDS (LR-MDS) are to manage anemia, improve or maintain quality of life (QoL), and delay disease progression to higher-risk MDS (HR-MDS) and acute myeloid leukemia (AML) [1, 2]. Chronic anemia in LR-MDS is associated with increased mortality and morbidity rates [3, 4].

Regular red blood cell (RBC) transfusions are used to manage LR-MDS-associated anemia despite their association with poorer QoL and significant cost; long-term RBC transfusions can lead to iron overload [1]. Erythropoiesis-stimulating agents (ESAs) are first-line therapy to treat anemia in patients with LR-MDS with serum erythropoietin (EPO) levels ≤500 U/l and low transfusion burden [2, 5]. However, the median duration of response to ESAs is 6–19 months, and >67% of patients with LR-MDS are, or become, refractory to ESAs after exposure [6, 7].

Luspatercept, a first-in-class erythroid maturation agent, was approved by the US Food and Drug Administration and European Medicines Agency to treat anemia in transfusion-dependent patients with ring sideroblast (RS)-positive LR-MDS who are refractory/intolerant to ESAs, following the phase 3 MEDALIST study (NCT02631070) [8–10]. A greater proportion of patients treated with luspatercept than placebo experienced RBC transfusion independence (RBC-TI) ≥8 weeks and RBC-TI ≥16 weeks during weeks 1–24 and 1–48 [8, 11]. The most common treatment-emergent adverse events (TEAEs) associated with ≤48 weeks of luspatercept treatment included fatigue, diarrhea, asthenia, nausea, and dizziness [8]; except for dizziness and diarrhea, the exposure-adjusted rates were either comparable with placebo or lower in the luspatercept arm [12]. Four patients (3 receiving luspatercept) progressed to AML during the study [8]. Luspatercept efficacy beyond 48 weeks and its impact on overall survival (OS) have not been reported. Here we present over 2 years of additional safety and efficacy data with concomitant impact on OS for MEDALIST trial patients.

The phase 3, double-blind, placebo-controlled MEDALIST trial [8] evaluated luspatercept for treatment of anemia in adults with Revised International Prognostic Scoring System [13] Very low-, Low-, or Intermediate-risk RS-positive MDS, who were refractory, intolerant, or unlikely to respond to ESAs, and were receiving regular RBC transfusions. Patients were randomized 2:1 to luspatercept (1.0 mg/kg up to 1.75 mg/kg) or placebo, administered subcutaneously every 3 weeks. The primary endpoint was RBC-TI for ≥8 weeks during weeks 1–24. Following completion of the study, patients who continued to benefit from luspatercept were eligible to enter long-term follow-up and remained on their prior dose and schedule regime. Patients receiving placebo received supportive care during follow-up. This analysis presents pooled data from two data cutoffs: November 26, 2020 (MEDALIST final parent protocol data cutoff) and January 15, 2021 (long-term follow-up data cutoff).

**Fig. 1:**
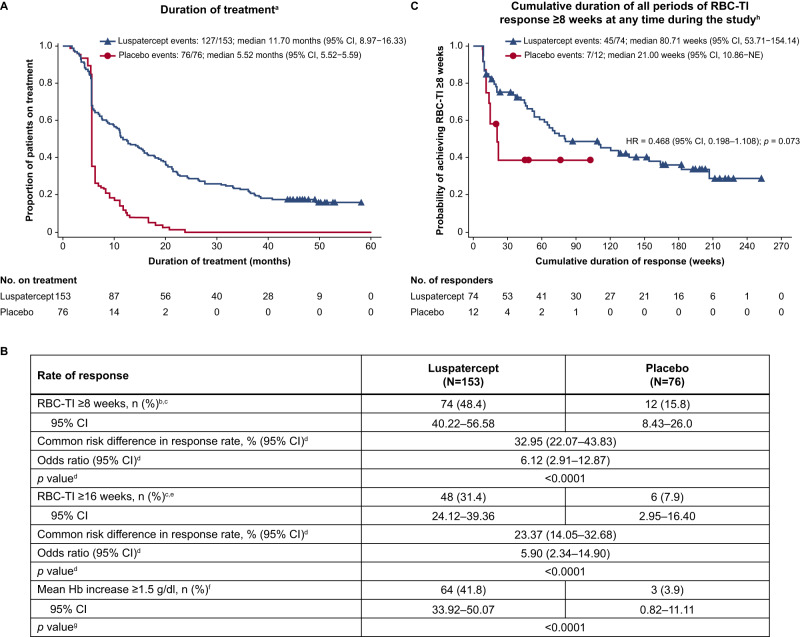
Duration of treatment, cumulative duration of RBC-TI ≥8 weeks response, and rates of achievement of RBC-TI ≥8 weeks and ≥16 weeks, and mean Hb increase ≥1.5 g/dl. **A** Duration of treatment during the entire treatment period. **B** Rate of achievement of RBC-TI ≥8 weeks and ≥16 weeks, and mean Hb increase ≥1.5 g/dl during the entire treatment period. **C** Cumulative duration of all periods of RBC-TI response ≥8 weeks at any time during the study. ^a^Treatment end date is defined as the last dose date +20 days, study discontinuation date, cutoff date or death date, whichever is earlier. ^b^Response is defined as the absence of any RBC transfusion during any consecutive 56-day period during the entire treatment period. ^c^Response rate (%) was calculated using the number of responders divided by the number of patients multiplied by 100. ^d^Cochran-Mantel-Haenszel test stratified for average baseline RBC transfusion requirement (≥6 units vs. <6 units of RBC per 8 weeks) and baseline IPSS-R score (Very low or Low vs. Intermediate). ^e^Response is defined as the absence of any RBC transfusion during any consecutive 112-day period during the entire treatment period. ^f^Defined as the proportion of patients with a Hb increase ≥1.5 g/dl compared with baseline that was sustained over any consecutive 56-day period in the absence of RBC transfusions. ^g^Fisher exact test comparing the luspatercept arm with the placebo arm. ^h^The cumulative duration of all periods of RBC-TI ≥8 weeks is defined as the sum of all durations of non-consecutive periods of RBC-TI response ≥8 weeks for patients during the entire treatment phase. ^i^Median is from the Kaplan–Meier method. CI confidence interval, Hb hemoglobin, HR hazard ratio, IPSS-R Revised International Prognostic Scoring System, NE not estimable, RBC red blood cell, RBC-TI RBC transfusion independence.

Baseline characteristics for MEDALIST patients (previously reported [8]) are included in Supplementary Table S1. At the last cutoff, the median follow-up time for patients in the luspatercept arm was 39.9 months (vs. 38.4 months for placebo), and the median duration of treatment was 11.70 months (vs. 5.52 months for placebo; Fig. 1A).

During the entire treatment period (as of January 15, 2021), RBC-TI ≥8 weeks was experienced by 74 of 153 (48.4%) patients receiving luspatercept and 12 of 76 (15.8%) patients receiving placebo (*p* <0.0001; Fig. 1B). Kaplan–Meier estimates of the cumulative duration of all periods of RBC-TI ≥8 weeks response, even if non-consecutive, for patients at any time during the study were 80.71 (95% confidence interval [CI]: 53.71–154.14) weeks with luspatercept and 21.00 (95% CI: 10.86–not estimable [NE]) weeks with placebo (hazard ratio [HR]: 0.468 [95% CI: 0.198–1.108]; *p* = 0.073; Fig. 1C). RBC-TI ≥16 weeks was experienced by 48 of 153 (31.4%) and 6 of 76 (7.9%) patients treated with luspatercept and placebo (*p* <0.0001), respectively (Fig. 1B). Overall, 64 of 153 (41.8%) patients receiving luspatercept experienced a mean increase in hemoglobin (Hb) ≥1.5 g/dl in the absence of any RBC transfusions compared with 3 of 76 (3.9%) patients receiving placebo (*p* <0.0001; Fig. 1B).

Overall rates of progression to HR-MDS and AML were low in both treatment arms: 9 of 153 (5.9%) patients receiving luspatercept and 3 of 76 (3.9%) of those receiving placebo progressed to HR-MDS, and 4 of 153 (2.6%) and 3 of 76 (3.9%) patients treated with luspatercept and placebo, respectively, progressed to AML. Follow-up time adjusted incidence rates of progression to HR-MDS per 100 person-years were 2.22 (95% CI: 1.16–4.27) and 1.55 (95% CI: 0.50–4.82) for patients treated with luspatercept and placebo, and 0.97 (95% CI: 0.37–2.60) and 1.52 (95% CI: 0.49–4.72), respectively, for patients who progressed to AML (Supplementary Table S2). Baseline characteristics of the 7 patients who progressed to AML are reported in Supplementary Table S3. No patients receiving luspatercept who progressed to AML also progressed to HR-MDS, compared with 1 patient receiving placebo who progressed to both HR-MDS and AML.

**Fig. 2:**
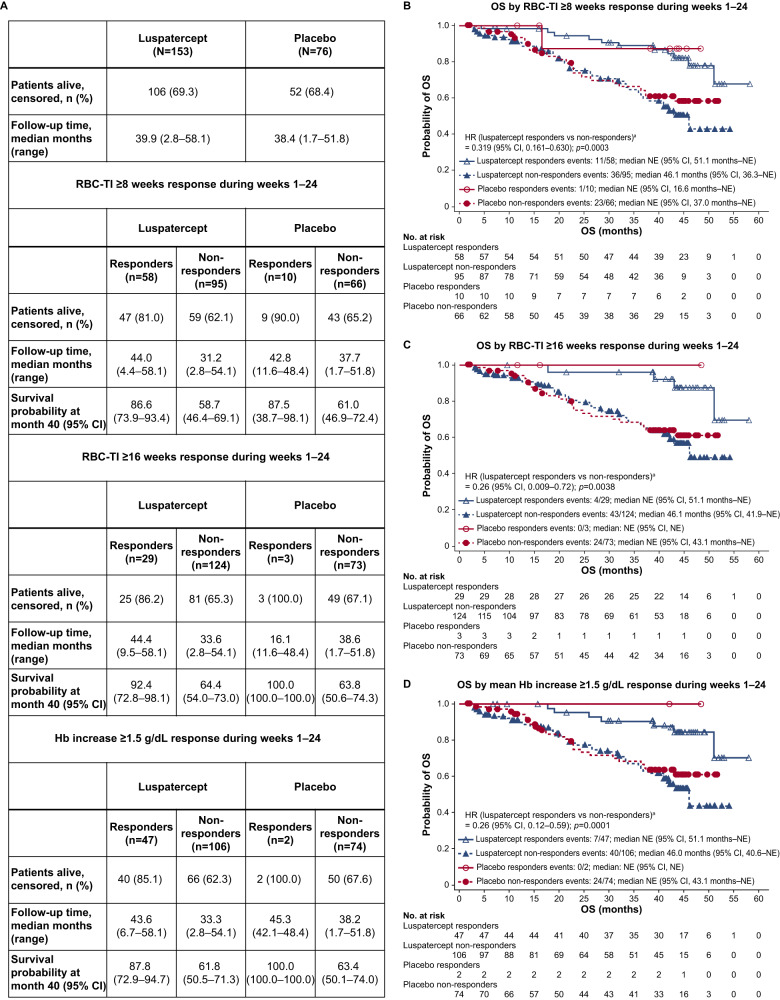
OS by RBC-TI ≥8 weeks response, RBC-TI ≥16 weeks response, and mean Hb increase ≥1.5 g/dl response. **A** Summary of OS. **B** OS by RBC-TI ≥8 weeks response during weeks 1–24. **C** OS by RBC-TI ≥16 weeks response during weeks 1–24. **D** OS by mean Hb increase ≥1.5 g/dL response during weeks 1–24. OS is calculated as the time from randomization date to death of any cause and is censored at the last date that the patient was known to be alive for patients who were alive at the time of analysis and for patients who discontinued from the study or were lost to follow-up. ^a^Unstratified HR and log-rank test *p* value. CI confidence interval, Hb hemoglobin, HR hazard ratio, NA not applicable, NE not estimable, OS overall survival, RBC-TI red blood cell transfusion independence, SD standard deviation.

As of January 15, 2021, 106 of 153 (69.3%) patients treated with luspatercept and 52 of 76 (68.4%) treated with placebo were alive (Fig. 2A); the median duration of OS was not reached by patients receiving luspatercept (95% CI: 46.1 months–NE) nor placebo (95% CI: 43.1 months–NE; Supplementary Fig. S1). Response to luspatercept during weeks 1–24 of the MEDALIST study was associated with longer OS. Of the 58 patients receiving luspatercept who experienced RBC-TI ≥8 weeks, 47 (81.0%) were alive compared with 59 of 95 (62.1%) patients who did not (Fig. 2A). The median duration of OS for luspatercept responders who experienced RBC-TI ≥8 weeks was not reached (95% CI: 51.1 months–NE) and was 46.1 (95% CI: 36.3–NE) months for non-responders (HR: 0.32 [95% CI: 0.16–0.63]; *p* = 0.0003; Fig. 2B). Of the 29 patients receiving luspatercept who experienced RBC-TI ≥16 weeks, 25 (86.2%) were alive compared with 81 of 124 (65.3%) patients who did not (Fig. 2A). The median duration of OS for luspatercept patients who experienced RBC-TI ≥16 weeks not reached (95% CI: 51.1 months–NE) compared with 46.1 (95% CI: 41.9–NE) months for non-responders (HR: 0.26 [95% CI: 0.09–0.72]; *p* = 0.0038; Fig. 2C). Of the 47 patients treated with luspatercept who experienced a mean Hb increase ≥1.5 g/dl in the absence of RBC transfusions, 40 (85.1%) were alive versus 66 of 106 (62.3%) patients who did not (Fig. 2A). The median duration of OS for patients receiving luspatercept who experienced a mean Hb increase ≥1.5 g/dl was not reached (95% CI: 51.1 months–NE) and was 46.0 (95% CI: 40.6–NE) months for non-responders (HR: 0.26 [95% CI: 0.12–0.59]; *p* = 0.0005; Fig. 2D).

We report that patients on the MEDALIST study continue to benefit from long-term luspatercept treatment over 2 years, with a median follow-up time of 39.9 months (vs. 38.4 months for placebo) compared with 26.4 months (vs. 26.1 months for placebo) from a previous report of longer-term luspatercept clinical benefit [11]. Compared to the first 24 weeks of the MEDALIST study [8], a greater proportion of patients who continued luspatercept treatment long-term experienced RBC-TI ≥8 weeks (48% vs. 38%) and RBC-TI ≥16 weeks (31% vs. 19%). The median longest duration of RBC-TI ≥8 weeks response in the primary MEDALIST analysis was 30.6 weeks [8]. In the current analysis, the median cumulative duration of RBC-TI response with luspatercept treatment was 80.7 weeks, consistent with the previous finding that 79% of MEDALIST primary endpoint responders experienced multiple separate RBC-TI ≥8 weeks response periods [12]. Taken together, these data demonstrate that patients continue to benefit from luspatercept beyond the first 24-week assessment period.

The MEDALIST study was not powered for an analysis of OS, however, this long-term analysis shows that, while median OS duration was similar between the luspatercept and placebo arms, achievement of response (RBC-TI ≥8 weeks, RBC-TI ≥16 weeks, or a mean Hb increase ≥1.5 g/dl during weeks 1–24) was associated with longer OS. One-quarter to one-third of LR-MDS cases will progress to AML [14]. In the primary MEDALIST analysis, 4 (1.7%) patients progressed to AML (including 3 [2.0%] patients receiving luspatercept) and 1 patient in each arm progressed to HR-MDS [8]. In the current analysis, rates of AML progression were similarly low, with 4 (2.6%) patients in the luspatercept arm and 3 (3.9%) in the placebo arm progressing to AML. Of note, patients who received luspatercept had a longer median time to AML progression from original MDS diagnosis versus placebo (61.70 vs. 32.69 months). Rates of HR-MDS progression were also low, with 9 (5.9%) patients receiving luspatercept and 3 (3.9%) receiving placebo progressing to HR-MDS. No new safety concerns with long-term luspatercept administration were identified in the current analysis.

In conclusion, these data demonstrated that long-term luspatercept treatment of transfusion-dependent anemia in patients with RS-positive LR-MDS provided sustained clinical benefit with a predictable safety profile, and RBC-TI ≥8 weeks, RBC-TI ≥16 weeks, and/or an increase in Hb ≥1.5 g/dl during the first 24 weeks of treatment was associated with improved OS among ESA-intolerant/refractory patients with RS-positive LR-MDS.

### Supplementary information

SUPPLEMENTARY APPENDIX

## Data Availability

BMS policy on data sharing may be found at https://www.bms.com/researchers-and-partners/independent-research/data-sharing-request-process.html.
