# Supplementary material for: Long-term utilization and benefit of luspatercept in transfusion-dependent, erythropoiesis-stimulating agent-refractory or -intolerant patients with lower-risk myelodysplastic syndromes with ring sideroblasts
Source: Leukemia. 2023 Sep 26;37(11):2314–8. doi: 10.1038/s41375-023-02031-7 (PMC10624606; doi:10.1038/s41375-023-02031-7)
Supplement: Supplementary file 1 — SUPPLEMENTARY APPENDIX [file 41375_2023_2031_MOESM1_ESM.docx]

**SUPPLEMENTARY APPENDIX**

**Methods**

**Table S1.** Baseline characteristics of MEDALIST trial patients.

| **Characteristic** | **Luspatercept**  **(*n* = 153)** | **Placebo**  **(*n* = 76)** |
| --- | --- | --- |
| Age,^a^ median, years (range) | 71.0 (40–95) | 72.0 (26–91) |
| Male sex, n (%) | 94 (61.4) | 50 (65.8) |
| Time since original MDS diagnosis,^b^ median, months (range) | 44.0 (3–421) | 36.1 (4–193) |
| Time since original MDS diagnosis categories,^b^ *n* (%) | | |
| ≤2 years | 40 (26.1) | 19 (25.0) |
| 2 to 5 years | 62 (40.5) | 34 (44.7) |
| >5 years | 51 (33.3) | 23 (30.3) |
| Ring sideroblasts ≥15%, *n* (%) | 153 (100) | 76 (100) |
| MDS WHO classification, *n* (%) | | |
| MDS RARS | 7 (4.6) | 2 (2.6) |
| MDS RCMD^c^ | 145 (94.8) | 74 (97.4) |
| Other^d^ | 1 (0.7) | 0 |
| IPSS-R classification risk category, *n* (%) | | |
| Very low | 18 (11.8) | 6 (7.9) |
| Low | 109 (71.2) | 57 (75.0) |
| Intermediate | 25 (16.3) | 13 (17.1) |
| High | 1 (0.7) | 0 |
| Serum EPO,^e^ median, U/l (range) | 156.9  (12–2454) | 130.8  (29–2760) |
| Serum EPO categories, *n* (%) | | |
| <100 U/l | 51 (33.3) | 31 (40.8) |
| 100 to <200 U/l | 37 (24.2) | 19 (25.0) |
| 200 to 500 U/l | 43 (28.1) | 15 (19.7) |
| >500 U/l | 21 (13.7) | 11 (14.5) |
| Missing | 1 (0.7) | 0 |
| Mutated *SF3B1*, *n* (%) | 141 (92.2) | 65 (85.5) |
| Baseline RBC transfusion burden, RBC units/8 week over period of 16 weeks,^f^ median (range) | 5 (1–15) | 5 (2–20) |
| Baseline RBC transfusion-burden category, *n* (%) | | |
| ≥6 units/8 weeks | 66 (43.1) | 33 (43.4) |
| 4 to <6 units/8 weeks | 41 (26.8) | 23 (30.2) |
| <4 units/8 weeks | 46 (30.1) | 20 (26.4) |
| Pretransfusion Hb level,^g^ median, g/dl (range) | 7.6 (6–10) | 7.6 (5–9) |
| Platelet count, median, × 10^-9^/l (range) | 235.0 (59–892) | 222.5 (60–689) |
| Received ESA previously, n (%) | 148 (96.7) | 70 (92.1) |
| Disease refractory to ESA, n/N (%) | 144/148 (97.3) | 69/70 (98.7) |
| Previous iron chelation therapy, n (%) | 71 (46.4) | 40 (52.6) |

CI confidence interval, EPO erythropoietin, ESA erythropoiesis-stimulating agents, Hb hemoglobin, IQR interquartile range, MDS myelodysplastic syndromes, RARS refractory anemia with RS, RBC red blood cell, RBC-TI RBC transfusion independence, RCMD refractory cytopenia with multilineage dysplasia, RS ring sideroblasts, SD standard deviation, WHO World Health Organization.

^a^Age was calculated based on the informed consent signing date.

^b^Time since original MDS diagnosis was defined as the number of years from the date of original diagnosis to the date of informed consent.

^c^All patients were classified as RCMD-RS as they were required to have RS per inclusion criteria.

^d^Locally diagnosed MDS-RS and multilineage dysplasia.

^e^Baseline EPO was defined as the highest EPO value within 35 days of the first dose of the investigational product.

^f^The analysis included data only within the 16 weeks before randomization.

^g^The pretransfusion Hb level was defined as the last value measured on or before the date and time of the first dose.

**TABLE S2.** Progression to AML or HR-MDS.

|  | **Luspatercept**  **(*n* = 153)** | **Placebo**  **(*n* = 76)** |
| --- | --- | --- |
| Patients who progressed to AML, *n* (%) | 4 (2.6) | 3 (3.9) |
| Total person-years, years | 410.3 | 197.0 |
| Incidence rate per 100 person-years (95% CI)^a^ | 0.97 (0.37–2.60) | 1.52 (0.49–4.72) |
| Kaplan–Meier estimates | | |
| Median time to AML progression, months (95% CI)^b^ | NE (NE–NE) | NE (NE–NE) |
| *p* value^c^ | 0.5925 | |
| HR (95% CI)^d^ | 0.665 (0.148–2.988) | |
| Summary of follow-up times, months | | |
| Median (IQR) | 39.16 (18.86–44.22) | 38.19 (15.82–44.71) |
| Time to AML progression from original MDS diagnosis, months | | |
| Median (IQR)^e^ | 61.70 (56.97–144.87) | 32.69 (30.13–60.35) |
| Patients who progressed to HR-MDS, *n* (%) | 9 (5.9) | 3 (3.9) |
| Total person-years, years | 405.2 | 193.1 |
| Incidence rate per 100 person-years (95% CI)^a^ | 2.22 (1.16–4.27) | 1.55 (0.50–4.82) |
| Kaplan–Meier estimates | | |
| Median time to HR-MDS progression, months (95% CI)^b^ | NE (NE–NE) | NE (NE–NE) |
| *p* value^c^ | 0.5457 | |
| HR (95% CI)^d^ | 1.496 (0.402–5.569) | |
| Summary of follow-up times, months | | |
| Median (IQR) | 38.70 (18.86–44.22) | 37.75 (15.34–44.29) |
| Time to HR-MDS progression from original diagnosis, months | | |
| Median (IQR)^f^ | 48.99 (33.77–64.89) | 53.49 (31.05–219.27) |

AML acute myeloid leukemia, CI confidence interval, HR hazard ratio, HR-MDS high-risk myelodysplastic syndromes, IQR interquartile range, IPSS-R Revised International Prognostic Scoring System, NE not estimable, RBC red blood cell, SD standard deviation, WHO World Health Organization.

^a^Person-year is calculated from randomization date to AML or HR-MDS onset date, or to last follow-up date for patients without progression to AML or HR-MDS.

^b^Median is from the Kaplan–Meier method.

^c^*p* value from log-rank test to compare luspatercept and placebo stratified by average baseline RBC transfusion requirement (≥6 units vs. <6 units of RBC per 8 weeks), and baseline IPSS-R score (Very low or Low, vs. Intermediate).

^d^The hazard ratio is from the Cox proportional hazards model with RBC transfusion requirement (≥6 units vs. <6 units of RBC per 8 weeks) and baseline IPSS-R (Very low or Low, vs. Intermediate) as covariates.

^e^Time to AML progression is defined as the time between randomization and first diagnosis of AML as per WHO classification of ≥20% blasts in peripheral blood or bone marrow.

^f^Time to HR-MDS progression is defined as the time between randomization and first diagnosis of HR-MDS.

**TABLE S3.** Baseline characteristics of patients who progressed to AML during the entire treatment period.

| **Characteristic** | **Luspatercept (n = 4)** | | | | **Placebo (n = 3)** | | |
| --- | --- | --- | --- | --- | --- | --- | --- |
| Patient | 1 | 2 | 3 | 4 | 1 | 2 | 3 |
| AML grade | 3 | 3 | 3 | 3 | 4 | 3 | 4 |
| WHO subtype | MDS RCMD | MDS RCMD | MDS RCMD | MDS RCMD | MDS RCMD | MDS RCMD | MDS RCMD |
| IPSS-R | Low | Low | Intermediate | Low | Low | Low | Low |
| Baseline mutations | *SF3B1*, *GATA2*, *RUNX1* | *DNMT3A*, *TET2*, *SF3B1* | *SF3B1*, *DNMT3A*, *TET2* | *SF3B1*, *DNMT3A*, *TET2* | *SF3B1* | *SF3B1*, *TET2* | *SF3B1* |
| Time from diagnosis to treatment (y) | 18.2 | 1.6 | 4.6 | 5 | 1 | 2.1 | 2.1 |
| Baseline TB (RBC units/8 weeks) | 4 | 6 | 10 | 8 | 2 | 3.5 | 5.5 |
| Serum ferritin (µg/L) | 749.5 | 914.7 | 2639.5 | 2147.5 | 1421 | 1379.5 | 1772.5 |
| Serum EPO (U/l) | 32.28 | 308.95 | 325.28 | 182.19 | 1056.52 | 54.04 | 90.23 |

**Fig. S1. OS for luspatercept versus placebo.**

**
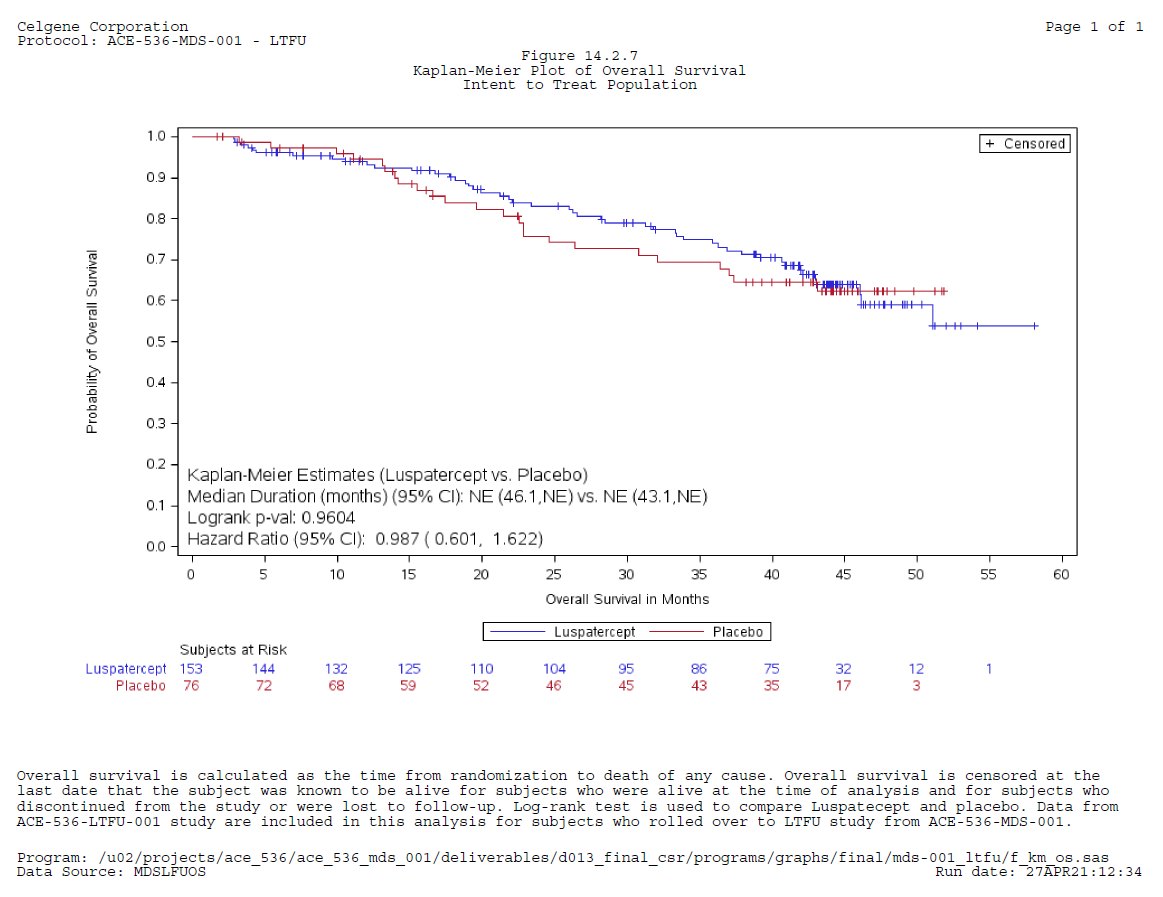
**

Data cutoff: January 15, 2021.

OS is calculated as the time from randomization to death of any cause. OS is censored at the last date the patient was known to be alive for patients who were alive at the time of analysis and for patients who discontinued from the study or were lost to follow-up. Log-rank test stratified by average baseline RBC transfusion requirement (≥6 units vs. <6 units of RBC per 8 weeks), and baseline IPSS-R score (Very low or Low, vs. Intermediate) is used to compare luspatercept and placebo. CI confidence interval, HR hazard ratio, IPSS-R Revised International Prognostic Scoring System, NE not estimable, OS overall survival, RBC red blood cell,
